# Synergistic Effect of High Charge and Energy Particle Radiation and Chronological Age on Biomarkers of Oxidative Stress and Tissue Degeneration: A Ground-Based Study Using the Vertebrate Laboratory Model Organism *Oryzias latipes*

**DOI:** 10.1371/journal.pone.0111362

**Published:** 2014-11-06

**Authors:** Xuan Zheng, Xinyan Zhang, Lingling Ding, Jeffrey R. Lee, Paul M. Weinberger, William S. Dynan

**Affiliations:** 1 Department of Neuroscience and Regenerative Medicine, Georgia Regents University, Augusta, Georgia, United States of America; 2 Center for Gene Diagnosis, Zhongnan Hospital, Wuhan University, Wuhan, China; 3 Department of Biostatistics, University of Alabama at Birmingham, Birmingham, Alabama, United States of America; 4 Department of Anatomy and Embryology, Wuhan University School of Medicine, Wuhan, China; 5 Department of Pathology, Georgia Regents University, Augusta, Georgia, United States of America; 6 Department of Otolaryngology and Center for Biotechnology & Genomic Medicine, Georgia Regents University, Augusta, Georgia, United States of America; 7 Departments of Radiation Oncology and Biochemistry, Emory University, Atlanta, Georgia, United States of America; National Research Council of Italy, Italy

## Abstract

High charge and energy (HZE) particles are a main hazard of the space radiation environment. Uncertainty regarding their health effects is a limiting factor in the design of human exploration-class space missions, that is, missions beyond low earth orbit. Previous work has shown that HZE exposure increases cancer risk and elicits other aging-like phenomena in animal models. Here, we investigate how a single exposure to HZE particle radiation, early in life, influences the subsequent age-dependent evolution of oxidative stress and appearance of degenerative tissue changes. Embryos of the laboratory model organism, *Oryzias latipes* (Japanese medaka fish), were exposed to HZE particle radiation at doses overlapping the range of anticipated human exposure. A separate cohort was exposed to reference γ-radiation. Survival was monitored for 750 days, well beyond the median lifespan. The population was also sampled at intervals and liver tissue was subjected to histological and molecular analysis. HZE particle radiation dose and aging contributed synergistically to accumulation of lipid peroxidation products, which are a marker of chronic oxidative stress. This was mirrored by a decline in PPARGC1A mRNA, which encodes a transcriptional co-activator required for expression of oxidative stress defense genes and for mitochondrial maintenance. Consistent with chronic oxidative stress, mitochondria had an elongated and enlarged ultrastructure. Livers also had distinctive, cystic lesions. Depending on the endpoint, effects of γ-rays in the same dose range were either lesser or not detected. Results provide a quantitative and qualitative framework for understanding relative contributions of HZE particle radiation exposure and aging to chronic oxidative stress and tissue degeneration.

## Introduction

Exposure to fast-moving atomic nuclei, known as high charge and energy (HZE) particles, is a limiting factor in the design of exploration-class human space missions, defined as those that venture beyond low earth orbit and thus beyond the partial protection afforded by the earth's magnetic field [1]. Prior work has shown that HZE particle radiation is associated with cancer and a number of other aging-like phenomena, including atherosclerosis, bone loss, and cognitive or behavioral impairment (for examples see [2]–[10]; reviewed in [11]).

Persistent oxidative stress is suspected to be an underlying factor in many of these degenerative effects observed at the organ level (reviewed in [12], [13]. Elevated levels of reactive oxygen species or their reaction products have been measured following HZE particle radiation exposure of both cells and animals [14]–[18]. Although HZE particles appear to be particularly effective, other DNA damaging agents also induce oxidative stress. Possible mechanisms leading to generation or release of reactive oxygen species include mitochondria injury [19], [20], DNA damage-mediated activation of NADPH oxidases [21], [22], and p53-mediated repression of PGC-1α, a master regulator of mitochondrial function and antioxidant gene expression [23]. Oxidative stress propagates from cell to cell, in part by signaling mechanisms [16], [24], and is a target for countermeasure development [14], [25].

The primary objective of the present study was to evaluate the increment in persistent oxidative stress due to radiation exposure, relative to the natural increase that occurs during aging. This is particularly challenging at the low doses representative of anticipated human exposure. Recent data from the Mars Science Laboratory suggest that a human round-trip mission to Mars would incur a physical radiation dose of about 0.16 Gy and an equivalent dose of about 0.66 Sv [26]. The large-scale, lifetime study described here was designed to address the challenges of measuring the potentially small effect of radiation doses in this range against a substantial age-dependent background. Secondary objectives of the study were to investigate potential mechanisms underlying increased oxidative stress through characterization of radiation-associated changes in gene expression, histology, and mitochondrial ultrastructure.

We performed studies using a vertebrate model organism, *Oryzias latipes* (the Japanese medaka fish). Medaka and other teleost models, such as the zebrafish, occupy a unique experimental niche. They have many vertebrate-specific organs of radiobiological interest and homologs of most or all of the DNA damage response and repair genes found in humans and other mammals. Most laboratory fish species also undergo regular, time-dependent aging similar to that in mammals. Importantly for the present study, it is feasible to maintain large numbers of individuals for lifetime studies at a cost much lower than for rodents.

The medaka species has a nearly 50-year history of use in radiation research [27]–[29]. The embryos tolerate a wide range of temperatures and can be chilled to delay development [30], which facilitates the logistics of beam-line studies [31]. We have previously investigated markers of normal aging in the medaka and shown that the liver is one of the first organs to show age-dependent degenerative changes [32]. The liver is also target of HZE carcinogenesis in the mouse [3], making it a logical focus for the study of HZE effects.

Our results show that a single exposure to HZE particle radiation, in a dose range overlapping that of anticipated human exposure, significantly elevates the levels of lipid peroxidation products in liver, when measured months or years following initial irradiation. The effect of radiation is synergistic with normal aging. A single developmental exposure to HZE particle radiation exposure is also associated with abnormal mitochondrial ultrastructure, cystic degeneration, and persistently decreased levels of expression of PPARGC1A, a master regulator of mitochondrial and antioxidant gene expression. Effects on the various endpoints were either lesser or not measurable with γ-rays in the same dose range, indicating that many of these endpoints are radiation quality dependent.

## Materials and Methods

### Animal maintenance

This study was carried out in strict accordance with the recommendations in the Guide for the Care and Use of Laboratory Animals of the National Institutes of Health. The protocol (number BR09-10-259) was approved by the Institutional Animal Care and Use Committee at Georgia Regents University. (Office of Laboratory Animal Welfare Assurance number A3307-1). The experiments did not involve procedures accompanied by pain, distress, or discomfort for which analgesics or anesthetics would be indicated.

Breeding stocks of CAB wild type Japanese medaka (*Oryzias latipes*) were maintained as described [32]. Embryos were collected and incubated for 24 h in aerated 0.67X seawater (3.5% NaCl) containing 0.0001% methylene blue. Viable (unstained) embryos were transferred to embryo rearing medium (0.1% NaCl, 0.003% KCl, 0.004% CaCl_2_ dihydrate, 0.016% Mg_2_SO_4_ heptahydrate, 0.001%, NaHCO_3_, pH 7.4) and held at 18°C with daily medium exchanges. Embryos collected over a 5 d period were pooled and shipped at ambient temperature to the NASA Space Radiation Laboratory in Brookhaven, New York. Embryos were re-staged according to established criteria [33] and stage 22–34 embryos were pooled and re-distributed randomly among six dose groups, including one mock-irradiated control group (n≈250 per group). Based on our prior study of normal aging [32], this group size provides 80% power to detect a 10% difference in mean survival assuming a two-sided α of 0.05.

Embryos were exposed, at mid-day, to 1 GeV/nucleon ^56^Fe ions at 0.1–0.4 Gy/min in T-25 flasks filled with embryo rearing medium. The primary method of calibrating the dose at NSRL is via a calibration ion chamber that is periodically calibrated using a standard gamma ray source. Before the exposures, the calibration ion chamber was used to measure the dose at the same time as an in-line secondary ion chamber was read. This reading served to transfer the calibration to the secondary ion chamber, which remained in the beam during the exposures. The secondary ion chamber was used to measure the integrated dose delivered and to cut off the beam when the desired total dose was reached. To facilitate experimental logistics, groups were exposed in order from highest to lowest dose, which allowed time for decay of activation products in the higher dose groups prior to returning embryo-containing flasks from the beam line facility to the biology laboratory.

After 48 h, embryos were shipped from Brookhaven to the home laboratory. They were maintained with a 12 h∶12 h light:dark photoperiod at ambient temperature in ∼200 ml aerated embryo rearing medium, which was changed daily. Every 2–3 d, newly hatched fry were transferred to containers containing continuously aerated tank water. They were fed twice daily with Zeigler Aquatox feed (Aquatic Ecosystems) passed through a 106 µm mesh filter. After two months, fry were transferred to a rack habitat system (Aquatic Habitats, Apopka, FL). Fish were maintained in conditioned water with quality parameters as follows: pH 7.5–8.3; conductivity, 500–560 µS; alkalinity, 80–100 mg/L as CaCO_3_; hardness, 100–120 mg/L as CaCO_3_; and dissolved oxygen, 5–7 mg/L. Fish were fed freely until they reached satiation twice daily, once with brine shrimp plus flake food in the morning and once with flake food in the afternoon.

A parallel γ-ray exposure arm of the experiment was performed at the home laboratory using a Gammacell Exactor (MDS Nordion, Ottawa, ON, Canada) at 0.8 Gy/min. Timed exposures were performed, with radiation dose estimated based on the manufacturer's calibration as adjusted for decay. We previously verified the accuracy of the calibration of this instrument using thermoluminescence dosimetry devices (Landauer Inc., Glenwood, IL, USA). [34]. There were seven dose groups, including one mock-irradiated control group. Embryos were maintained thereafter as for the HZE-irradiated cohort.

Survival was scored prior to hatching based on exclusion of methylene blue dye and after hatching by direct observation of motility. The condition of the fish was monitored at least once daily. Fish were humanely euthanized if they displayed an inability to ambulate (swim) properly or weight loss in excess of 15% of body weight.

### Tissue processing and staining

At 250, 400, 500, and 600 d post-irradiation, eight male fish were randomly chosen from each dose group (except for the 27 Gy γ-ray group, where only four male survivors remained). As in previous work, we evaluated livers in male fish only [32], because the female liver, which produces vitellogenin as part of oogenesis, is highly variable between individuals. Fish were euthanized with 400 mg/L Tricaine (MS-222) and livers were divided into portions for histologic fixation and RNA extraction. Fixation was performed in Bouin's solution for 48 h. Samples were rinsed in 50% ethanol and kept in 70% ethanol at 4°C until they lost their yellow color. After paraffin embedding, 5 µm sections were stained with hematoxylin and eosin and evaluated blindly by a pathologist. For anti-4-hydroxy-2-nonenal (4-HNE) staining, sections were deparaffinized, rehydrated, and stained as described [32] using 1.1 ng/µl anti-4-HNE primary antibody (HNE-J2, Japan Institute for the Control of Aging, Fukuroi, Japan) and 1∶500 biotinylated goat anti-mouse IgG secondary antibody (Vector Laboratories). Immune complexes were visualized using VECTASTAIN ABC standard and ImmPACT DAB kits (Vector Laboratories) with hematoxylin counterstaining. Where indicated, primary antibody was neutralized with 4-HNE-BSA (Cell Biolabs, San Diego, CA, USA). DAB staining was quantified using a Nuance multispectral imaging system 3.0.0 (Cambridge Research and Instrumentation Inc. Woburn, MA, USA). Spectral profiles were defined using a slide without primary antibody to define hematoxylin and a slide with high DAB intensity to define DAB plus hematoxylin. Subsequently a pure DAB spectral profile was calculated from the hematoxylin and the DAB plus hematoxylin profiles. Images were captured at 20 nM intervals from 480 to 720 nM, and spectrally unmixed. The hematoxylin component image was used to create an investigator-defined region of interest (ROI) mask, which was then applied to the DAB-specific component image. Nuance values (in optical density, OD) were recorded for the DAB component only, as average signal per ROI as a continuous scalar variable.

### Polymerase chain reaction

For mRNA analysis, total liver RNA was extracted using Trizol and PureLink RNA Mini Kit (Ambion by Life Technologies, Grand Island, NY). Reverse transcription using a QuantiTect Reverse Transcription Kit (Qiagen, Valencia, CA) was performed to obtain cDNA. We identified medaka orthologs of various genes of radiobiological interest based on their annotation in the ENSEMBL genome browser (www.ensembl.org) and verified them based on alignment to other full-length vertebrate homologs. Primers were designed such that at least one member of each pair spanned an mRNA splice junction (Table S1). The qPCR reactions were performed using a QuantiFast SYBR Green PCR kit (Qiagen, Valencia, CA). The inability to amplify genomic DNA, which may be present as a contaminant in RNA samples, was verified experimentally. Specificity of the PCR products was verified by gel electrophoresis and melting curve analysis. The ΔΔC_t_ method was used to determine fold change between treatment groups. Normalization was performed using the geometric average of two reference genes, ACTB (β-actin) and RPL7 [35], [36].

### Electron microscopy

Liver tissue was fixed in 2% glutaraldehyde in 0.1 M sodium cacodylate (NaCac) buffer, pH 7.4, postfixed in 2% osmium tetroxide in NaCac, stained *en bloc* with 2% uranyl acetate, dehydrated with a graded ethanol series and embedded in Epon-Araldite resin. Thin sections were cut with a diamond knife on a Leica EM UC6 ultramicrotome (Leica Microsystems, Inc., Bannockburn, IL), collected on copper grids and stained with uranyl acetate and lead citrate. Cells were observed in a JEM 1230 transmission electron microscope (JEOL USA Inc., Peabody, MA) at 110 kV and imaged with an Ultras can 4000 CCD camera & First Light Digital Camera Controller (Gating Inc., Pleasanton, CA).

### Statistical methods

For 4-hydroxynonenal (4-HNE), we attempted to fit to a general linear model using age and dose as predictors and 4-HNE scores as the outcome. The homogeneity assumption was not met, and a logarithmic transformation of the outcome was therefore applied. HZE particle radiation and γ-ray cohorts were analyzed separately. For RNA analysis, we again fitted to a general linear model using age and dose as predictors and ΔΔC_t_ values as the outcome. For the HZE cohort, assumptions were met for the PPARGC1A, SOD2, CDKN1A, SIRT3, and PTGES genes. For the γ-ray cohort, assumptions were met for the PPARGC1A, CAT, CDKN1A, and PTGES genes. For analysis of mitochondrial ultrastructure, differences in area were evaluated by ANOVA and then by a Dunnett's test comparing different dose groups with the non-irradiated control. The fraction of abnormal mitochondria was evaluated by a Chi-square test. For necrotic cysts, data from different time points were pooled and evaluated by ordinal logistic regression. In all cases the unit of analysis was single animals or tissue samples derived from single animals.

## Results

### Experimental design

**Figure 1:**
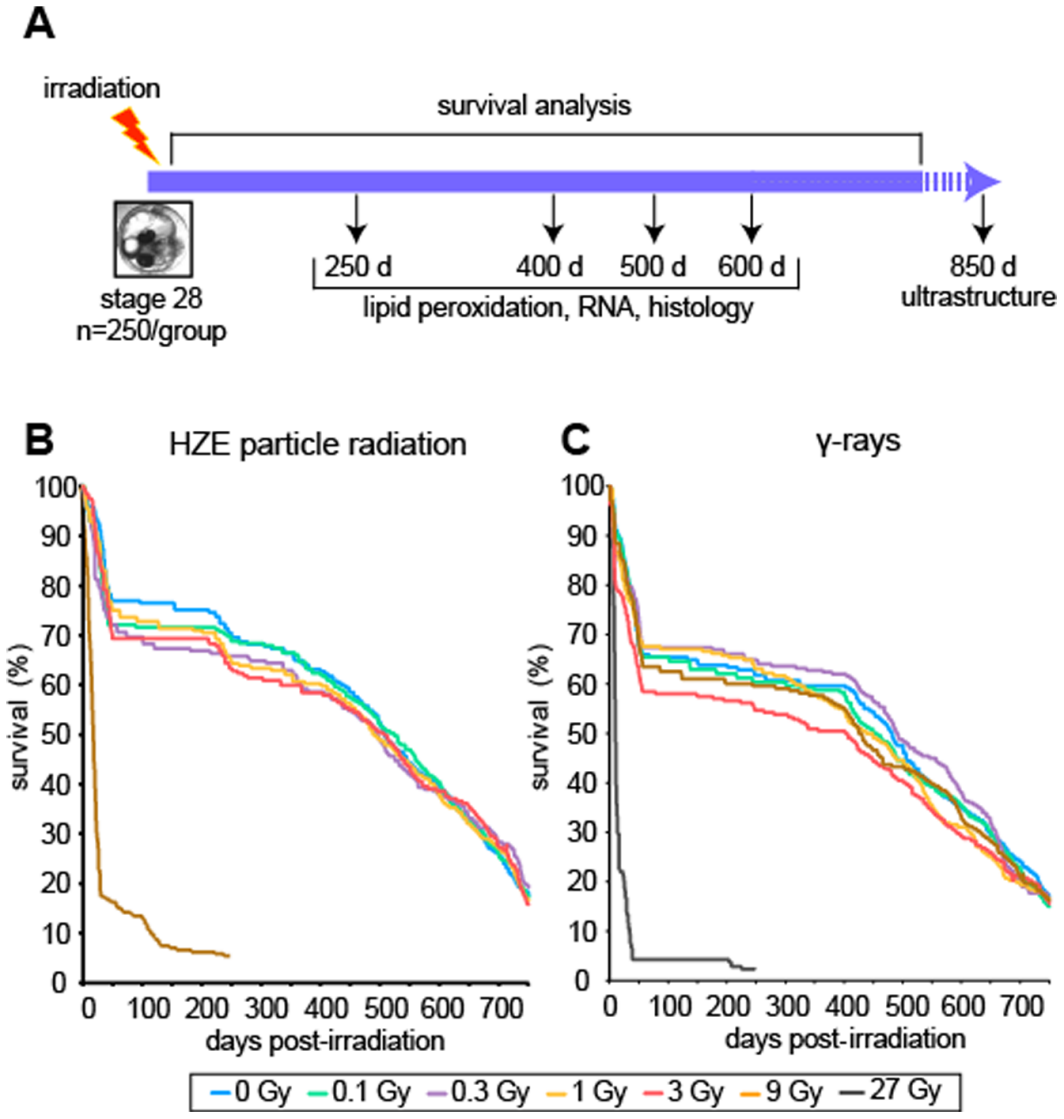
Experimental design and radiation survival. A. Embryos were irradiated with 1 GeV/u ^56^Fe ions or with γ-rays, reared, and scored for survival as described in Materials and Methods. At the indicated times, male fish from each dose group were sacrificed and the livers analyzed. B, C. Kaplan-Meier survival curves for fish exposed as embryos to indicated doses and types of radiation.

The experimental design was inspired by an early study of X-ray effects [37] and is summarized in Figure 1A. Medaka embryos were staged and randomized among experimental groups as described in Materials and Methods. Pools of embryos received a single exposure to HZE particle radiation or to reference γ-rays (n≈250 per dose group). Irradiation with HZE particles (1000 MeV/u ^56^Fe nuclei) was performed at the National Space Radiation Laboratory in Brookhaven, New York, using a range of doses from 0.1 Gy to 9.0 Gy in 3-fold increments. A separate cohort was irradiated with γ-rays using a ^137^Cs irradiator, using a range of doses from 0.1 Gy to 27 Gy in 3-fold increments. Populations were then observed and sampled at intervals until the median lifespan was exceeded. Measured endpoints included survival, growth, and histological and molecular changes in the liver.

### Effect of radiation exposure on growth and survival

Following irradiation, embryos were allowed to hatch out and survival was monitored for a minimum of 750 days, until about 80% mortality had been reached in all groups, including the non-irradiated controls. There was acute mortality in the 9 Gy HZE group and in the 27 Gy γ-ray group. All of the lower dose groups showed mortality curves similar to those for the non-irradiated controls. We observed some normal peri-hatching mortality, a plateau in survival that lasted about one year, and a period of steady decline in the number of survivors that lasted until the end of the experiment (Figures 1B and 1C). The median lifespan for individuals that survived the peri-hatching period was in the 500–550 day range, which is similar to that observed previously for medaka reared in our laboratory [32].

We did not observe gross developmental abnormalities or changes in swimming or feeding behavior in any of the sublethally irradiated groups. There were also no statistically significant effects of sublethal irradiation on weight or length of individuals sampled at intervals from the population (Figure S1).

### Effect of age and radiation exposure on a marker of persistent oxidative stress

Given the prior findings that HZE particle exposure is associated with oxidative stress, we hypothesized that outwardly normal individuals might nevertheless exhibit changes in oxidative stress-related biomarkers. At 250, 400, 500, and 600 days post-irradiation, we sampled eight individuals from each dose group and harvested livers for histologic and molecular analysis.

**Figure 2:**
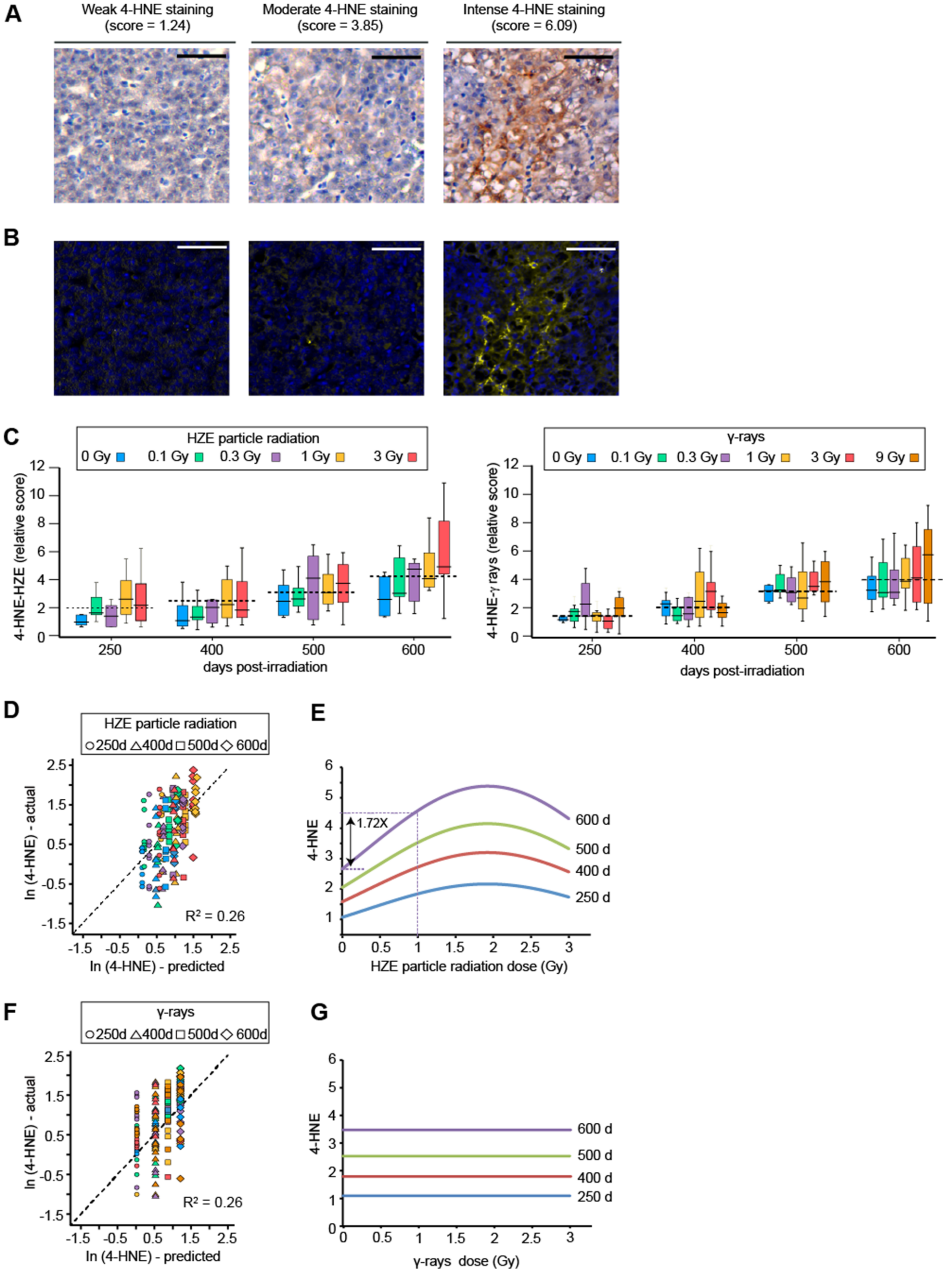
Age and dose-dependent accumulation of 4-hydroxynonenal in liver tissue. Liver sections were stained with anti-4-hydroxy-2-nonenal (4-HNE) and hematoxylin counterstain as described in Materials and Methods. A. Bright-field images showing weak, moderate, or intense anti-4-HNE immunohistochemical staining. Scale bars  = 20 µm. B. Nuance renderings of images from Panel A (yellow, 4-HNE; blue, cell nuclei). C. Quantification of 4-HNE staining based on Nuance analysis. Box plot shows mean and interquartile ranges. Color denotes dose group. Values are normalized to 0 Gy, 250 day group. D. Plot showing correlation between actual and predicted natural logarithm (ln)-transformed 4-HNE values for HZE cohort. Predicted values are based on regression model incorporating age, dose, and dose-squared parameters. Each symbol represents one individual. Shape denotes age group; color denotes dose group using same values as in Panel B. E. Plot showing predicted 4-HNE values as a function of age and HZE dose. F, G. Same as Panels D, E for γ-ray cohorts. Regression model incorporates an age parameter only; dose parameters were non-significant.

We evaluated levels of 4-hydroxy-nonenal, an end product of lipid peroxidation and a biomarker of chronic oxidative stress. We performed quantitative immunohistochemistry as described in Materials and Methods. Representative primary images of liver sections from control and irradiated individuals are shown in Figure 2A. The staining was specific for 4-HNE, as evidenced by the absence of staining when the primary antibody was omitted or when the primary antibody was neutralized with 4-HNE-BSA (Figure S2). For quantification, images were analyzed by Nuance multispectral analysis to separate anti-4-HNE staining from the hematoxylin counterstain. False-color renderings generated from the Nuance analysis are shown below the corresponding bright field images (Figure 2B).

Values obtained from the Nuance analysis are plotted in Figure 2C. Some trends are apparent in the raw data. Mean 4-HNE levels increased with age to a similar extent in both the HZE and γ-ray cohorts. In addition, mean 4-HNE levels were higher in all of the HZE-irradiated groups than in the corresponding age-matched control groups. To more rigorously evaluate age and radiation dose as predictors of 4-HNE levels, we performed multiple regression analysis. Residual values were not normally distributed and we therefore applied a logarithmic transformation. For the HZE particle radiation cohort, age and radiation dose were significant predictors. The best fit was obtained with a model of the form:
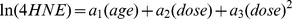
where a_1_, a_2_, and a_3_ are empirically determined. Parameter estimates, uncertainties, and *P* values are given in Table 1. To examine the goodness of fit, we analyzed the correlation between actual values of ln(4-HNE) versus those predicted by the model (Figure 2D). Based on the coefficient of determination (R^2^), the model explains 26% of the variation in the HZE cohort, and the residuals are randomly distributed.

**Table 1:** Age and dose dependence for 4-HNE.

	HZE			γ-ray		
Predictor	Parameter estimate	Parameter uncertainty	*P* value	Parameter estimate	Parameter uncertainty	*P* value
Intercept	−0.582	0.201	0.0044	−0.734	0.184	<0.0001
Age	0.0026	0.0004	<0.0001	0.0033	0.0004	<0.0001
Dose	0.735	0.228	0.0015	NS^a^	NS	NS
Dose squared	−0.191	0.072	0.0091	NS	NS	NS

a Not significant.

Units for age are days; units for dose are Gy. Parameters are for ln-transformed data.

The form of the model implies that the effects of age and dose are synergistic. That is, additive contributions of the age and dose terms to the logarithmically transformed 4-HNE values translate into multiplicative contributions to the real (non-transformed) 4-HNE values. To illustrate the relative contribution of age and dose in practical terms, we plotted 4-HNE values, as predicted by the model, as a function of dose (Figure 2E). In the example depicted in the figure, exposure to 1.0 Gy of HZE particle radiation increases 4-HNE levels by 1.72-fold. Note that because of the multiplicative relationship between age and radiation effects, the fold increment attributable to radiation is the same regardless of age.

We performed a similar analysis the γ-ray cohort. Age was a significant predictor but radiation dose was not. The best fit was thus obtained with a model of the form:
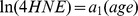



Parameter estimates, uncertainties, and *P* values are again given in Table 1. To examine the goodness of fit, we again analyzed the correlation between actual values of ln(4-HNE) versus those predicted by the model (Figure 2F). Based on the coefficient of determination (R^2^), the model explains 26% of the variation. To illustrate the model in practical terms, we again plotted 4-HNE levels versus dose (Figure 2G). Note that in this representation, the γ-ray model reduces to a series of parallel lines representing different age groups, as the radiation dose term is non-significant.

### Effect of age and radiation exposure on PPARGC1A and other mRNAs

It was of interest to investigate potential mechanisms underlying the elevated 4-HNE values in the HZE-exposed cohort. As one approach, we measured changes in mRNA levels for candidate genes that are involved in mitochondrial maintenance, antioxidant defense, growth control, or radiation-induced intercellular signaling. One of these was the medaka ortholog of PPARGC1A, which encodes a transcriptional coactivator, PGC-1α. In mammals, PGC-1α provides a unique link between the DNA damage response, metabolism, and oxidative stress [23]. It promotes expression of genes that are required for mitochondrial biogenesis and maintenance, and it coordinates expression of anti-oxidant defense genes. Declines in expression have been linked to aging and various disease states, reviewed in [38], [39]. Expression is also repressed by p53 as part of the chronic DNA damage response [23].

We hypothesized that a decline in PGC-1α expression might be a factor in radiation-induced oxidative stress. We designed primers specific for the medaka ortholog of PPARGC1A and performed qPCR using medaka ACTB (β-actin) and RPL7 as internal reference genes. Primer sequences and ENSEMBL transcript IDs for these and other genes in the study are shown in Table S1. All primer pairs yielded reverse-transcriptase PCR products showing a single sharp peak (Figure S3) on melting curve analysis.

**Figure 3:**
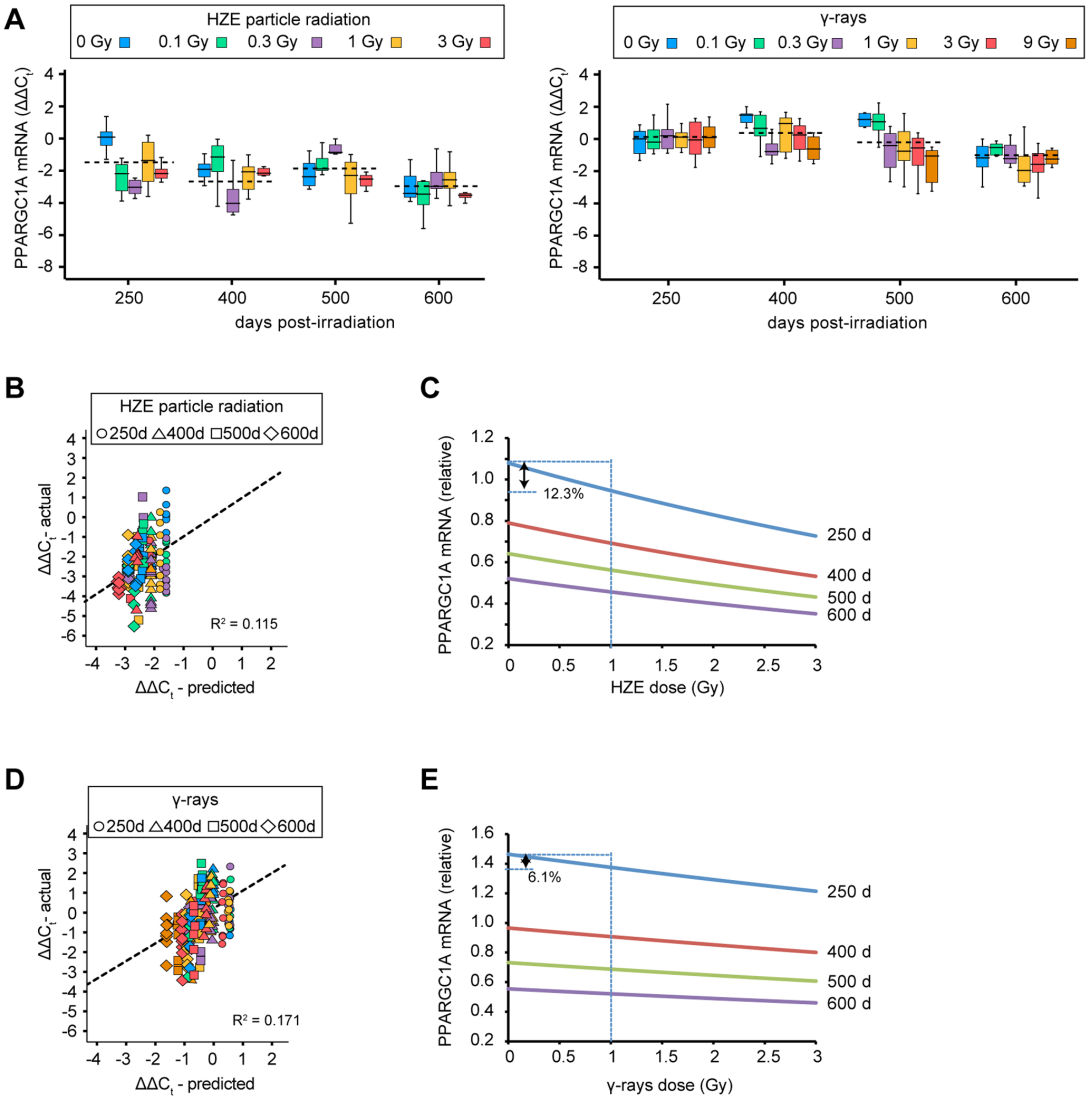
Age and dose-dependent decline in PPARGC1A mRNA in liver tissue. A. Quantification of PPARGC1A mRNA. Box plot shows mean and interquartile ranges. Color denotes dose group. Values are normalized to 0 Gy, 250 day group. B. Plot showing correlation between actual and predicted PPARGC1A values. Each symbol represents one individual. Shape denotes age group, color denotes dose group using same values as in Panel A. Plot showing predicted PPARGC1A values as a function of age and HZE dose. Note that vertical axis shows relative mRNA amounts (i.e., back-transformed from ΔΔC_t_ values). D, E, same as Panels B, C for γ-ray cohorts.

Relative PPARGC1A mRNA levels for HZE particle radiation and γ-ray cohorts are shown in Figure 3A. There was much more stochastic variation than with the 4-HNE data. However, multiple regression analysis showed that age and radiation dose were significant predictors of ΔΔC_t_ values for both the HZE and γ-ray cohorts. A model of the form:
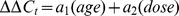
provided the best fit. As ΔΔC_t_ is related to logarithmically transformed mRNA abundance values, the model again implies a multiplicative, synergistic effect on real (non-transformed) mRNA abundance. Figure 3B and 3D show correlation plots for actual and predicted values for the HZE particle-irradiated and γ-ray cohorts, respectively. The models accounted for 11.5% and 17.1% of the experimental variation in the two cohorts. Parameters, uncertainties, and *P* values for the model are given in Table 2. As with the 4-HNE data, independently determined age parameters were similar for the HZE particle radiation and γ-ray cohorts. Because the dose parameters were significant for both cohorts, it was possible to estimate a nominal relative biological effectiveness (RBE) value of 2.1. This should, however, be interpreted with caution because of the large uncertainties in the parameter estimates.

**Table 2:** Age and dose dependence for expression of PPARGC1A mRNA.

	HZE			γ-ray		
Predictor	Parameter estimate	Parameter uncertainty	*P* value	Parameter estimate	Parameter uncertainty	*P* value
Intercept	−0.86	0.35	0.014	1.55	0.30	0.0001
Age	−0.003	0.0007	<0.0001	−0.004	0.0006	<0.0001
Dose	−0.19	0.09	0.03	−0.09	0.03	0.0005

Units for age are days; units for dose are Gy. Parameters are for ΔΔC_t_.

To illustrate the relative contributions of age and radiation dose, we plotted the predicted PPARGC1A mRNA levels as a function of dose (Figure 3C and 3E). The magnitude of the age effect was similar to that with 4-HNE, that is, a 2 to 4-fold change over the period from 250 to 600 days post exposure. The magnitude of the dose effect was much smaller, however. A 1 Gy dose of HZE or γ-rays leads to only a 12.3% or 6.1% decline in PPARGC1A expression, respectively. Thus, although a decrease in PPARGC1A may contribute to increased oxidative stress, it seems likely there may be other, unidentified genes to account for the magnitude of the oxidative stress phenotype.

In addition to PPARGC1A, we investigated the effects of age and radiation dose on several other genes that are involved in mitochondrial maintenance, anti-oxidant defense, growth control, or radiation-induced intercellular signaling (Figure S4). The largest effects of radiation dose were seen with CDKN1A, which encodes the p21 (WAF1/CIP1) protein, a potent cyclin-dependent kinase inhibitor. CDKN1A is a prototypical target of p53 activation and a marker of cell senescence (reviewed in [40], [41]). Expression of CDKN1A decreased (rather than increased) with dose in both the HZE particle radiation and γ-ray cohorts, with a nominal RBE of 3.7 (Figure S5). The results suggest that developmental exposure to radiation is associated with neither a chronic p53-dependent DNA damage response nor with widespread senescence. In contrast to PPARGC1A, the effects of age on CDKN1A expression were small and inconsistent between cohorts,

Other genes tested included SOD2 (superoxide dismutase) and CAT (catalase), which are oxidative stress defense genes; SIRT3 (Sirtuin 3), a mitochondrial histone deacetylase that is protective against aging and cellular damage (reviewed in [42]); and PTGES (prostaglandin E synthase), which catalyzes a step in biosynthesis of prostaglandin E2, a mediator of radiation bystander signaling that is induced at the protein level by HZE irradiation in mice [43]. In some cases, regression analysis showed significant effects of age or radiation dose (Table S2) although the effect sizes were small and the biological significance is uncertain.

### Change in mitochondrial morphology

The age and radiation-related increases in persistent oxidative stress seen in Figure 2, and the declines in PPARGC1A expression seen in Figure 3, led us to hypothesize that mitochondria might be compromised in HZE-irradiated individuals. To test this, we sampled the few long-term HZE radiation survivors that remained after other experiments were complete and prepared liver samples for ultrastructural analysis.

**Figure 4:**
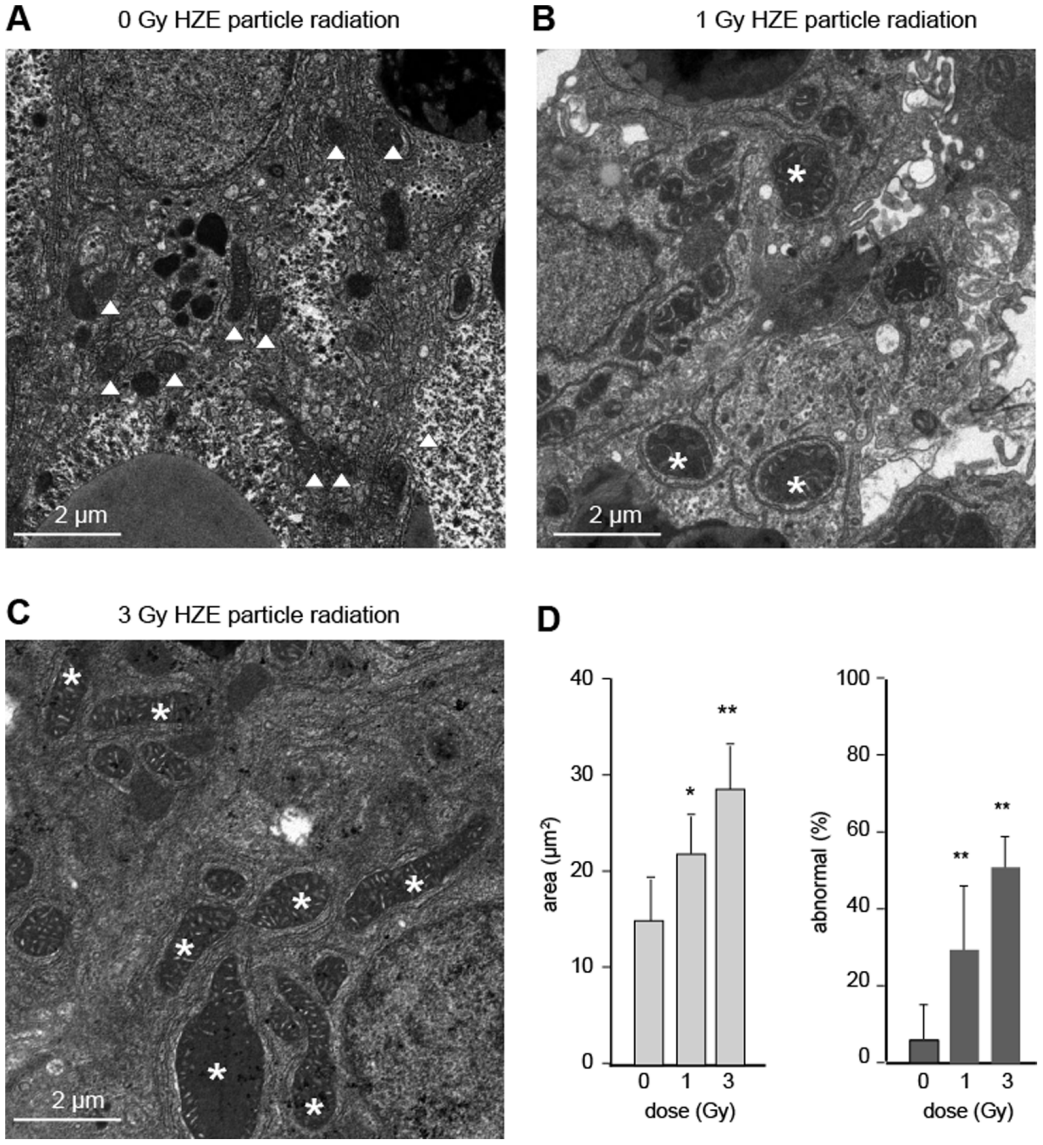
Analysis of mitochondrial ultrastructure. Panels A–C show relatively normal mitochondria (triangles) from a non-irradiated individual and two examples of enlarged and elongate mitochondria (*) from HZE-exposed groups. D. Mitochondrial area as a function of HZE dose (left panel). Percent of elongated mitochondria as a function of dose (right panel).

Three male fish each from the 0 Gy, 1 Gy, and 3 Gy groups were analyzed. Representative electron micrographs of liver sections are shown in Figure 4. Whereas samples from the 0 Gy groups showed predominantly normal mitochondrial size and shape (panel A), most of the samples from the 1 Gy and 3 Gy groups showed grossly enlarged or elongated mitochondria, sometimes with disorganized, circular cristae (panels B and C). Mitochondrial area was about 1.4-fold higher in the 1 Gy group and nearly 2-fold higher in the 3 Gy group, relative to non-irradiated controls (panel D). As an alternative method of analysis, we also determined the fraction of mitochondria with an area of more than 25 µm^2^. The 1 Gy and 3 Gy groups had a significantly higher fraction of these severely enlarged mitochondria (panel D).

### Presence of distinctive, necrotic cysts in livers from HZE cohort

We anticipated that radiation exposure might lead to liver cancer, particularly as the medaka liver is an established model for chemical carcinogenesis [44]. However, there were no histologically verified liver tumors in approximately 350 specimens examined. Indeed, among the 2000+ individuals observed in the lifetime study, only 2–3 animals were seen with grossly evident tumors. These sporadic tumors, which in most cases occurred near the eyes, were not analyzed further.

**Figure 5:**
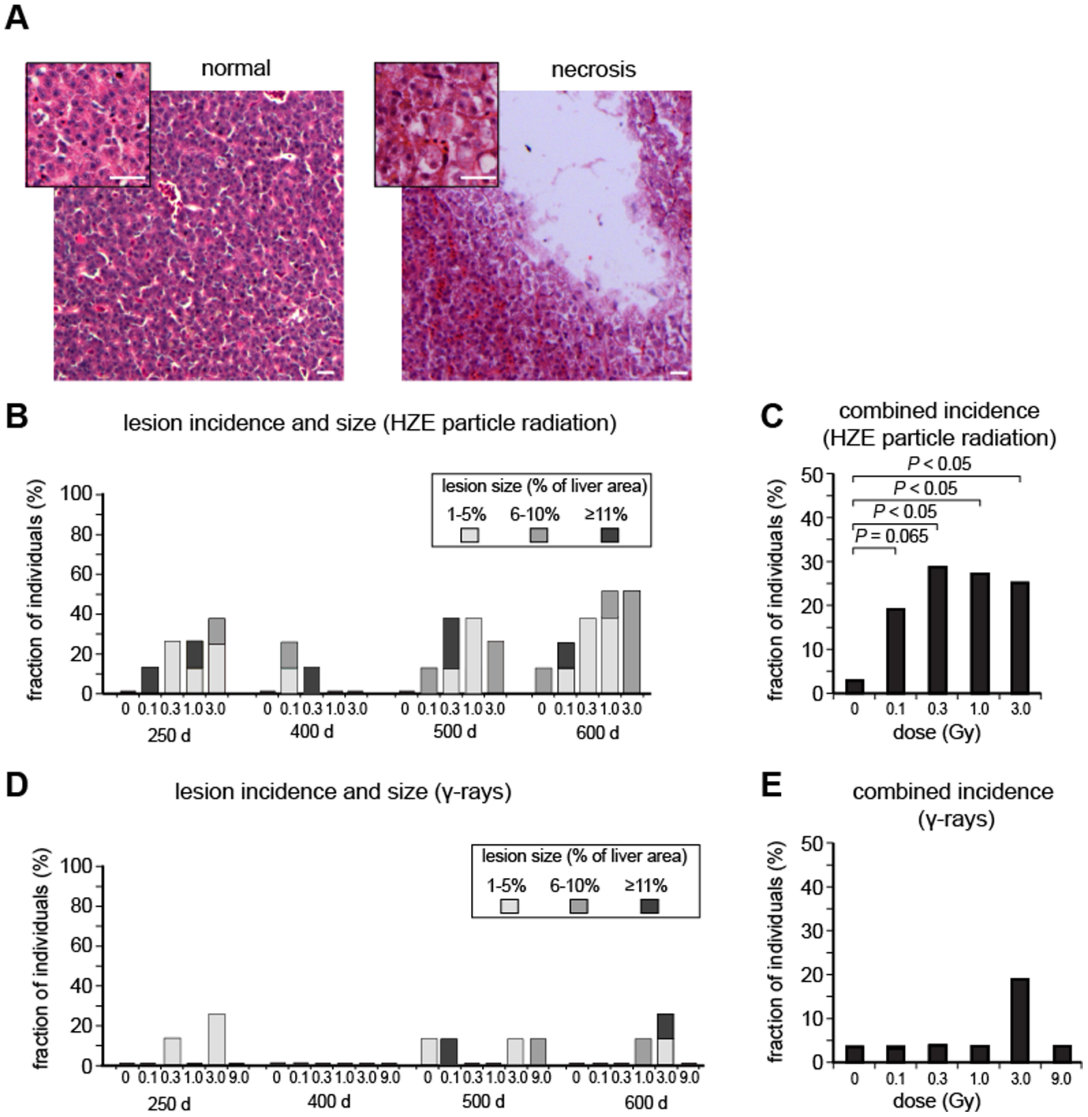
Necrotic cysts in livers of radiation-exposed individuals. A. Representative hematoxylin and eosin stained sections. The left panel shows normal liver, and the right panel shows a necrotic cyst. Insets show regions of each section at higher magnification. Scale bars are 20 µm. B. Stacked column graph showing the incidence of necrotic cysts, classified according to the percentage area of the liver that was affected. C. Pooled data showing incidence of necrotic cysts at different doses of HZE particle radiation. Lesions of different severity were combined and classified as abnormal. Different age groups were also combined. *P* values are shown based on ordinal logistic regression. D. Stacked column graph, as in Panel B but for γ-ray exposed groups. E. Pooled data showing incidence of necrotic cysts at different doses of γ-rays.

We did observe that about one third of the fish sampled for histology from the HZE-irradiated groups showed distinctive, necrotic cysts. Examples of these are shown in Figure 5A. Whereas normal liver is composed of a regular pattern of hepatocytes, separated by sinusoidal spaces, the cystic regions consisted of empty areas, up to 100 µm in diameter, surrounded by necrotic cells. A summary showing the incidence and severity of lesions in each HZE dose and age group are shown in Figure 5B. For statistical analysis, we pooled the data for different time points and for different severities and performed an ordinal logistic regression (Figure 5C). There was a significant increase over the non-irradiated controls at all doses ≥0.3 Gy. Above this threshold, incidence was dose-independent over a 10-fold range. Necrotic cysts were not present at significant levels in the γ-ray exposed groups (Fig. 5D and 5E).

Spongiosis hepatis is another type of cystic lesion that occurs spontaneously in adult medaka [32], [45]. It differs from the necrotic cysts in that the hepatic skeleton remains intact and the area surrounding the lesion remains normal. Some incidence of spongiosis hepatis was seen in most experimental groups, and in only one instance was the incidence significantly elevated relative to controls (0.3 Gy γ-rays) (Figure S5).

## Discussion

We report here the acute and long-term effects of HZE particle and γ-ray exposure in a laboratory model organism, the Japanese medaka fish. Acute mortality was seen only at the highest doses tested (9 Gy HZE particle radiation and 27 Gy γ-rays). Acute mortality following exposure to 27 Gy of γ-rays is consistent with prior reports of LD_50_ values of 20 and 26 Gy for low-linear energy transfer radiation in the medaka [31], [46].

In contrast to the acute effects, we found that long-term effects of HZE particle radiation were seen at lower doses, where a single exposure, early in life, led to a persistent increase in oxidative stress. Elevated levels of a quantifiable biomarker, 4-HNE, indicated that oxidative damage was present. Multiple regression analysis indicated synergy between radiation exposure and chronological age as predictors of 4-HNE levels. An additional, qualitative indicator of persistent oxidative stress was the abnormal mitochondrial ultrastructure observed in aged, HZE-exposed individuals. Mitochondrial homeostasis is maintained by a cycle of fission and fusion. In mammals, oxidative stress has been shown to perturb this cycle, leading to enlarged and elongated morphology [47]. In our HZE-exposed specimens, we observed similar, bizarrely elongated and enlarged mitochondria, evident more than two years after the original exposure.

The synergistic age and radiation-dependent decline in PPARGC1A mRNA may be one cause of the observed oxidative stress. PPARGC1A is a master regulator of genes involved in mitochondrial maintenance and defense against oxidative stress [48]. The decreased levels are suggestive of decreased mitochondrial function and inability to effectively detoxify reactive oxygen species. Of the candidate genes tested, the only other one to show such a marked, dose-dependent decline was CDKN1A, a classically TP53-inducible gene and a senescence marker. Although we did not have access to species cross-reactive antibodies that could be used to measure TP53 protein levels directly, the decline (rather than increase) in CDKN1A expression argues against the presence of activated p53 in the aging, irradiated populations. There were some other genes that showed significant radiation responses, although effect sizes were small and biological significance is uncertain.

Our best-fit model for the effect of HZE radiation exposure on 4-HNE levels assumed a nonlinear dose-response curve, that is, a curve that bends over at the highest doses, whereas the best-fit models for the PPARGC1A RNA data assumed a linear dose-response relationship. This apparent difference may or may not be biologically meaningful, as the radiation effect on PPARGC1A was smaller than the effect on 4-HNE, and we may not have had sufficient power to distinguish between linear and nonlinear models. The nonlinearity in the 4-HNE data primarily affects the predicted response at doses above the range of anticipated human exposure, and so may not be of practical significance.

We also observed distinctive necrotic cysts, but not liver cancer, in the HZE-exposed fish. They occurred in all HZE-exposed groups and did not show a significant dose response relationship. We did not see these lesions in non-irradiated fish or in the γ-ray cohort, or in our previous study of aging in the medaka [32]. While the cystic degeneration was a very notable feature of the HZE-exposed group, we do not at this time have a clear understanding of its etiology; perhaps it is an emergent phenomenon reflecting the various kinds of stress imposed by HZE exposure.

To investigate differences between the effects of HZE particle radiation and γ-rays, we exposed a separate cohort of medaka embryos to γ-radiation. It was necessary to establish the two cohorts sequentially, rather than simultaneously, because of the logistical difficulty in handling large numbers of embryos simultaneously. The γ-ray cohort was established about six weeks after the HZE cohort and was drawn from the same stocks. The two cohorts had approximately the same median lifespan and showed a similar age-dependent increase in 4-HNE levels and age-dependent decline in PPARGC1A mRNA. Thus, we regard them as biologically comparable. There was some radiation dependent increase in 4-HNE levels in the γ-ray cohort, but it was not statistically significant in the regression analysis. There were also radiation-dependent declines in PPARGC1A and CDKN1A, although these were smaller than for the HZE radiation-exposed cohort.

Quantitative and qualitative differences in HZE particle radiation and γ-rays presumably reflect the distinctive physics of tissue interaction. HZE particles produce a dense burst of reactive oxygen species along a nanometer-scale core track, whereas γ-rays, at the same dose, deposit energy along more numerous but less densely ionizing tracks. Dense ionization along the core HZE track leads to potentially irreparable DNA damage [12], [49]. At the same time, radial propagation of secondary electrons produces damage at sites elsewhere in the target cell or in neighboring cells. Thus, a single encounter with an HZE particle thus creates damage that is simultaneously denser and more widespread, potentially affecting DNA and the mitochondria simultaneously and in different ways. We speculate that this may produce a sufficient burst of damage from which cells never fully recover – an initial burst of reactive oxygen species leads to a self-perpetuating cycle of mitochondrial injury, leakage of endogenous reactive oxygen species, and further damage to mitochondria or other cellular components.

The medaka model has some limitations, notably the paucity of species cross-reactive antibodies. We were thus not able to measure levels of TP53 or phosphorylated ATM proteins, which would have provided direct information regarding the presence of a chronic DNA damage response. We were not able to measure 8-oxodeoxyguanosine, a major base oxidation product, because of high background staining. We were not able to detect γ-H2AX, a marker of unrepaired DNA double-strand breaks. It may be that examination of mRNA expression profiles in greater depth, for example by deep sequencing, will provide further information about the activation of DNA damage response pathways, but results of such studies are not available at the present time.

## Conclusions

A main rationale for animal studies of HZE particle radiation effects is the need to better understand human risk. Results here show that effects of HZE particle radiation can persist over the lifetime of an organism. These persistent effects occur at doses representative of the range of anticipated human exposure.

## Supporting Information

Figure S1 **Comparison of growth rates in different experimental groups.** Eight male fish were sampled from each dose group and time point. Colors denote dose as shown in key. A. HZE particle cohort, body length and body weight as indicated. B. γ-ray cohort, body length and weight as indicated.
(TIF)

Figure S2 **Controls for 4-HNE staining.** Liver sections were stained with anti-4-HNE and hematoxylin counterstain as described in Materials and Methods. Bright-field images are shown. Scale bars  = 20 µm. Left panel, staining under normal conditions; center panel, primary antibody omitted; right panel, primary antibody pre-incubated with 2 ng/µl 4-HNE BSA. Note absence of staining when primary antibody was omitted or pre-incubated with antigen.
(TIF)

Figure S3 **Melting curves for PCR products derived from primer pairs used in this study.** Melting curve analysis was performed for PCR products of each gene were analyzed, as indicated. Panels show fluorescence as a function of temperature. The first derivative of the melting curve is superimposed. Colors denote results with two independent samples. Note the sharp melting transition seen with each product. Genes are grouped by functional category as indicated.
(TIF)

Figure S4 **Quantification of candidate mRNAs other than PPARGC1A.** Box plot shows mean and interquartile ranges. Color denotes dose group as shown in key. Values are normalized to 0 Gy, 250 day group. Genes are grouped by functional category as indicated. Left panels, HZE particle radiation, right panels, γ-rays. Note that CDKN1A shows a decline in HZE particle-irradiated individuals (in almost all cases, irradiated groups show lower mean expression than age-matched control groups; see Table S2 for regression analysis). There was a smaller, but significant dose-dependent decline for γ-rays. Although age or dose were statistically significant predictors for some other genes, the magnitude of the effects were small and in some cases inconsistent between HZE radiation and γ-ray cohorts.
(TIF)

Figure S5 **Spongiosis hepatis in in livers of radiation-exposed individuals.** A. Representative hematoxylin and eosin stained section showing spongiosis hepatis; compare with normal and with necrotic cysts in Fig. 5 of main text. Inset shows region at higher magnification. Scale bars are 20 µm. B. Stacked column graph showing the incidence and size of regions of spongiosis in HZE particle radiation-exposed cohort. C. Pooled data showing incidence of spongiosis at different doses of HZE particle radiation. Lesions of different severity were combined and classified as abnormal. Different age groups were also combined. *P* values are shown based on ordinal logistic regression. D, E. Same as Panels B, C for γ-ray cohort.
(TIF)

Table S1 **Primer pairs used in this study.** Functional category, gene symbol, forward and reverse primer sequences, and ENSEMBL transcript identifiers are shown.
(PDF)

Table S2 **Age and dose dependence for expression of select mRNAs.** Table provides results of regression models based on quantification of mRNAs shown in Fig. S5. Category, gene symbol, parameter values and uncertainties, and *P* values are indicated. Parameter values are omitted where assumptions are violated for a univariate model.
(PDF)
