# Supplementary figures and images for: Synergistic Effect of High Charge and Energy Particle Radiation and Chronological Age on Biomarkers of Oxidative Stress and Tissue Degeneration: A Ground-Based Study Using the Vertebrate Laboratory Model Organism Oryzias latipes
Source: PLoS One. 2014 Nov 6;9(11):e111362. doi: 10.1371/journal.pone.0111362 (PMC4222877; doi:10.1371/journal.pone.0111362)

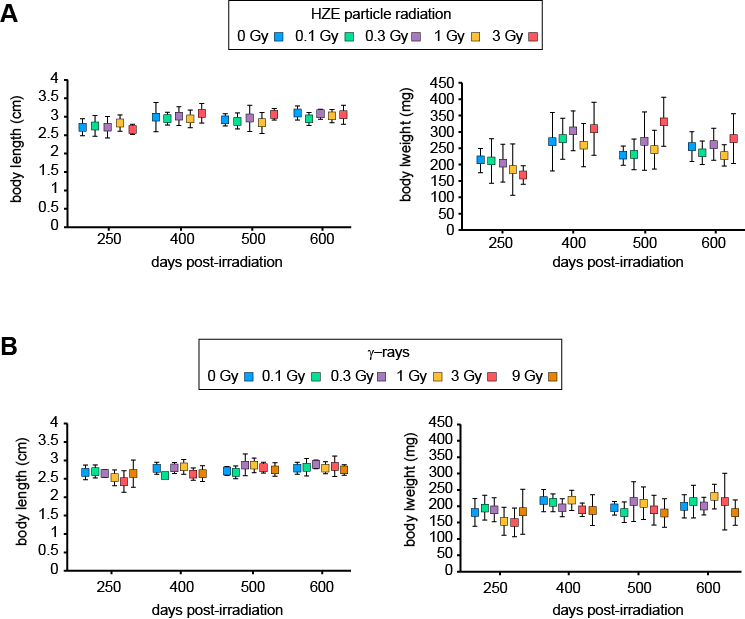

Supplement: Figure S1 — Comparison of growth rates in different experimental groups. Eight male fish were sampled from each dose group and time point. Colors denote dose as shown in key. A. HZE particle cohort, body length and body weight as indicated. B. γ-ray cohort, body length and weight as indicated. (TIF) [file pone.0111362.s001.tif]

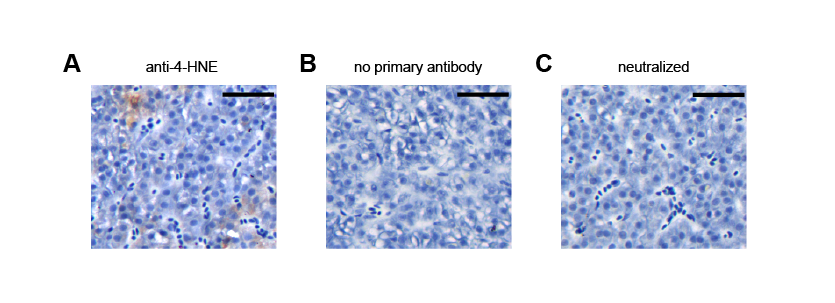

Supplement: Figure S2 — Controls for 4-HNE staining. Liver sections were stained with anti-4-HNE and hematoxylin counterstain as described in Materials and Methods. Bright-field images are shown. Scale bars = 20 µm. Left panel, staining under normal conditions; center panel, primary antibody omitted; right panel, primary antibody pre-incubated with 2 ng/µl 4-HNE BSA. Note absence of staining when primary antibody was omitted or pre-incubated with antigen. (TIF) [file pone.0111362.s002.tif]

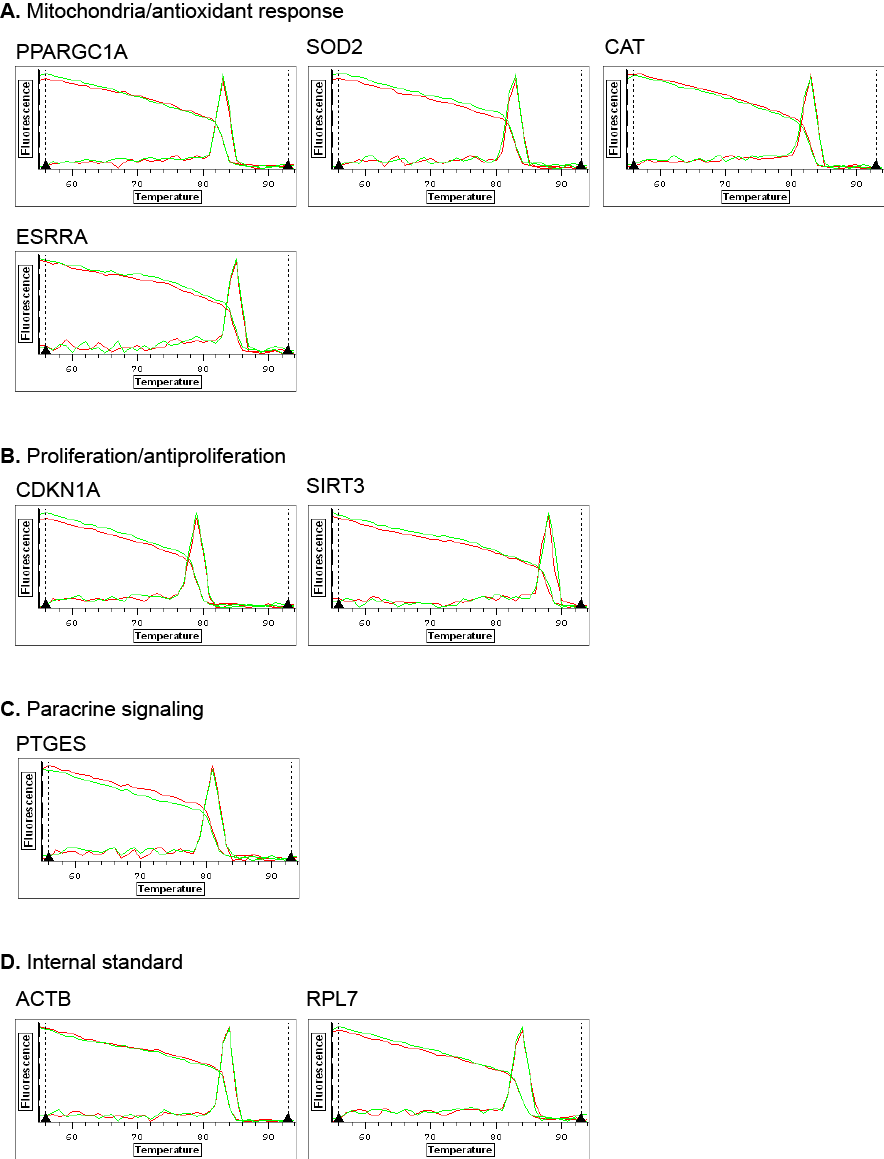

Supplement: Figure S3 — Melting curves for PCR products derived from primer pairs used in this study. Melting curve analysis was performed for PCR products of each gene were analyzed, as indicated. Panels show fluorescence as a function of temperature. The first derivative of the melting curve is superimposed. Colors denote results with two independent samples. Note the sharp melting transition seen with each product. Genes are grouped by functional category as indicated. (TIF) [file pone.0111362.s003.tif]

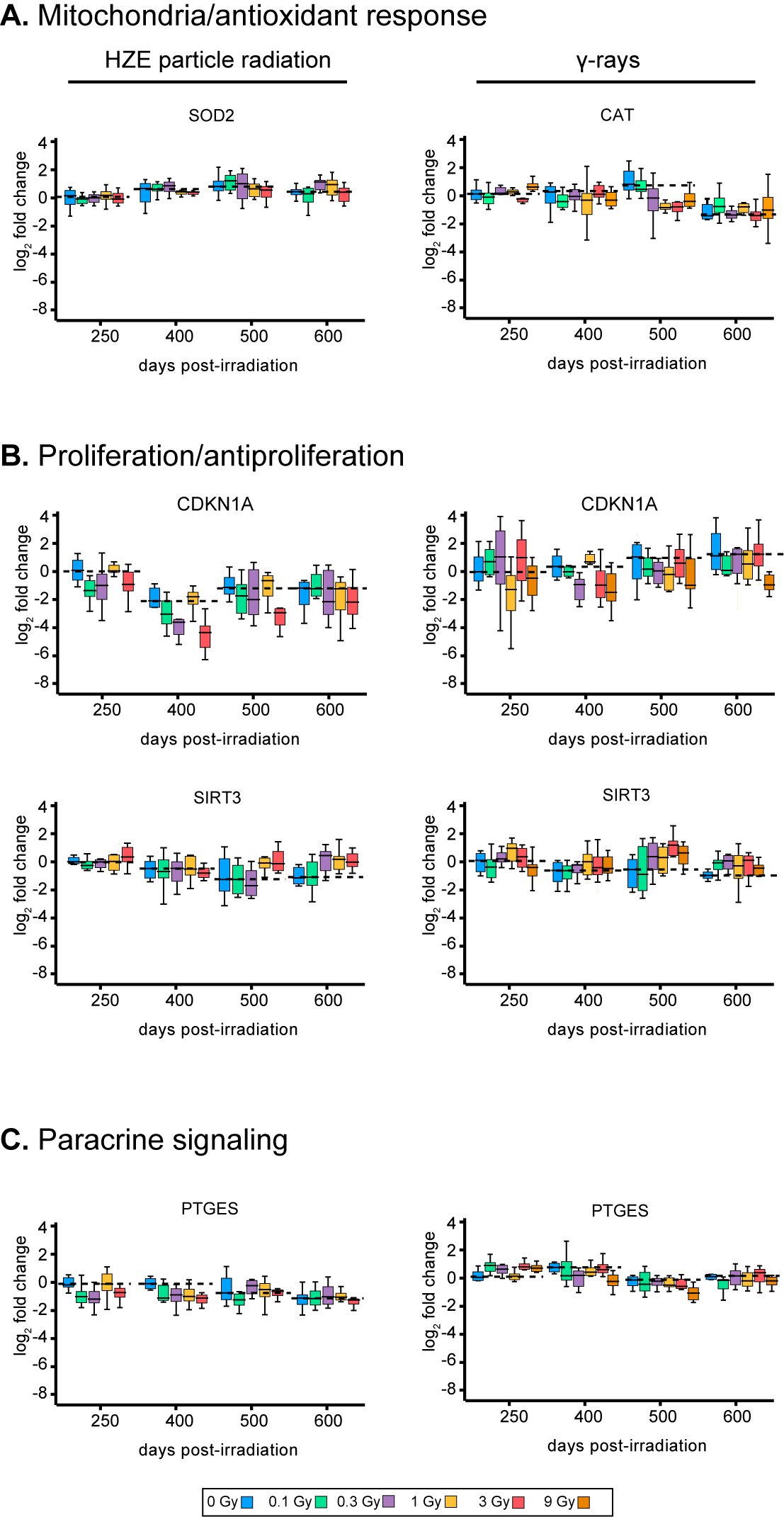

Supplement: Figure S4 — Quantification of candidate mRNAs other than PPARGC1A. Box plot shows mean and interquartile ranges. Color denotes dose group as shown in key. Values are normalized to 0 Gy, 250 day group. Genes are grouped by functional category as indicated. Left panels, HZE particle radiation, right panels, γ-rays. Note that CDKN1A shows a decline in HZE particle-irradiated individuals (in almost all cases, irradiated groups show lower mean expression than age-matched control groups; see Table S2 for regression analysis). There was a smaller, but significant dose-dependent decline for γ-rays. Although age or dose were statistically significant predictors for some other genes, the magnitude of the effects were small and in some cases inconsistent between HZE radiation and γ-ray cohorts. (TIF) [file pone.0111362.s004.tif]

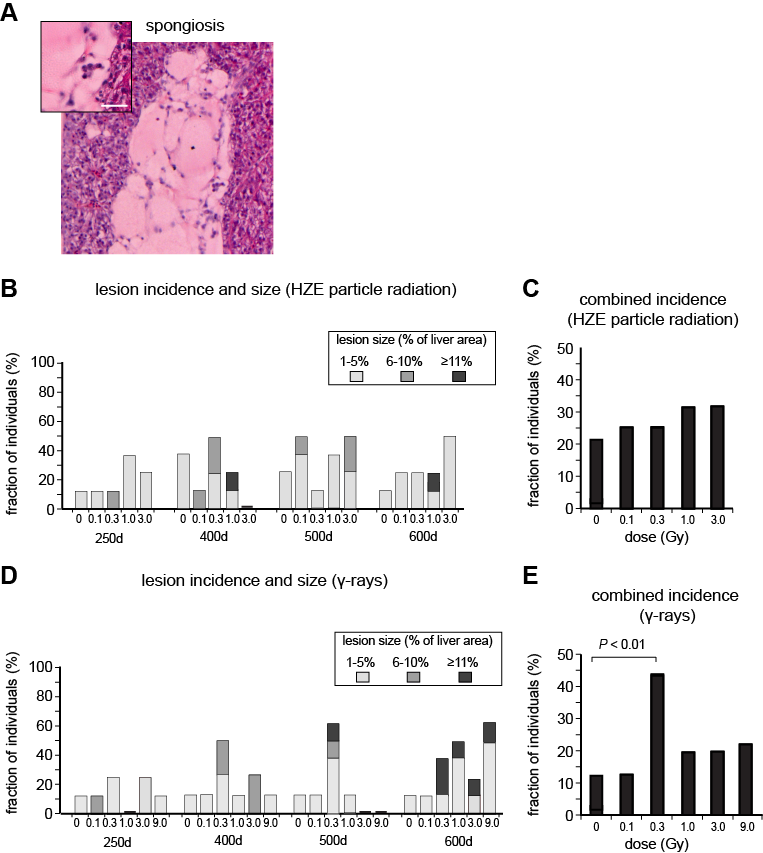

Supplement: Figure S5 — Spongiosis hepatis in in livers of radiation-exposed individuals. A. Representative hematoxylin and eosin stained section showing spongiosis hepatis; compare with normal and with necrotic cysts in Fig. 5 of main text. Inset shows region at higher magnification. Scale bars are 20 µm. B. Stacked column graph showing the incidence and size of regions of spongiosis in HZE particle radiation-exposed cohort. C. Pooled data showing incidence of spongiosis at different doses of HZE particle radiation. Lesions of different severity were combined and classified as abnormal. Different age groups were also combined. P values are shown based on ordinal logistic regression. D, E. Same as Panels B, C for γ-ray cohort. (TIF) [file pone.0111362.s005.tif]
