# Supplementary material for: Synergistic Effect of High Charge and Energy Particle Radiation and Chronological Age on Biomarkers of Oxidative Stress and Tissue Degeneration: A Ground-Based Study Using the Vertebrate Laboratory Model Organism Oryzias latipes
Source: PLoS One. 2014 Nov 6;9(11):e111362. doi: 10.1371/journal.pone.0111362 (PMC4222877; doi:10.1371/journal.pone.0111362)
Supplement: Table S1 — Primer pairs used in this study. Functional category, gene symbol, forward and reverse primer sequences, and ENSEMBL transcript identifiers are shown. (PDF) [file pone.0111362.s006.pdf]

Table S1

Functional category, gene symbol, sequences, and ENSEMBL transcript identifiers for qPCR primers

| Category | Gene | Primer sequence (5’-3’) | ENSEMBL transcript ID |
|---|---|---|---|
| Mitochondria/antioxidant response | PPARGC1A | TCAAGACGGAGAGCCCCTG<br>AGAGCCAATCCGCTCTTGTC | ENSORLT00000006028 |
| | SOD2 | TGACTGCACAGGTTACCCTT<br>TCCATCAGCTCCCCTTGCG | ENSORLT00000016614 |
| | CAT | CTACTGTTTCCGTCCTTCGT<br>CTCAGGCTCCAGAAGTCCCA | ENSORLT00000002176 |
| Proliferation/antiproliferation | CDKN1A | AACATCTTCAACGTGGAGAAA<br>GCTTGGTAGAAATCTGTGATGT | ENSORLT00000000343 |
| | SIRT3 | AGAGCTGACTCTGCAGGACATT<br>CTGCCCGGAGACCTGAAAT | ENSORLT00000000105 |
| Paracrine signaling | PTGES | GCAAGAAGGCTTTTGCCAA<br>CCAAGAAGAGGAAGGGTAAGATGT | ENSORLT00000004888 |
| Internal reference | ACTB | TCGCAGACAGAATGCAGAAGGAGA<br>ACTCCTGCTTGCTGATCCACATCT | ENSORLT00000017152 |
| | RPL 7 | TGTCATCAGGATCCGAGGTATCA<br>TACACCGTTGAAGATCTGGCG | ENSORLT00000009998 |
