# Supplementary material for: Synergistic Effect of High Charge and Energy Particle Radiation and Chronological Age on Biomarkers of Oxidative Stress and Tissue Degeneration: A Ground-Based Study Using the Vertebrate Laboratory Model Organism Oryzias latipes
Source: PLoS One. 2014 Nov 6;9(11):e111362. doi: 10.1371/journal.pone.0111362 (PMC4222877; doi:10.1371/journal.pone.0111362)
Supplement: Table S2 — Age and dose dependence for expression of select mRNAs. Table provides results of regression models based on quantification of mRNAs shown in Fig. S5. Category, gene symbol, parameter values and uncertainties, and P values are indicated. Parameter values are omitted where assumptions are violated for a univariate model. (PDF) [file pone.0111362.s007.pdf]

Table S2

Age and dose dependence for expression of select mRNAs

| Category | Gene | Predictor | HZE Parameter estimate | HZE Parameter uncertainty | HZE *P* value | γ-ray Parameter estimate | γ-ray Parameter uncertainty | γ-ray *P* value |
|---|---|---|---|---|---|---|---|---|
| | PPARGC1A | Intercept | -0.86 | 0.35 | 0.014 | 1.55 | 0.30 | 0.0001 |
| | | Age | -0.003 | 0.0007 | <0.0001 | -0.004 | 0.0006 | <0.0001 |
| | | Dose | -0.19 | 0.09 | 0.03 | -0.09 | 0.03 | 0.0005 |
| Mitochondria/antioxidant response | SOD2 | Intercept | -0.26 | 0.17 | 0.1255 | NS[a] | NS[a] | NS[a] |
| | | Age | 0.002 | 0.0004 | 0.0001 | NS[a] | NS[a] | NS[a] |
| | | Dose | -0.08 | 0.04 | 0.0445 | NS[a] | NS[a] | NS[a] |
| | CAT | Intercept | NS[a] | NS[a] | NS[a] | 0.83 | 0.23 | 0.0004 |
| | | Age | NS[a] | NS[a] | NS[a] | -0.003 | 0.0005 | <0.0001 |
| | | Dose | NS[a] | NS[a] | NS[a] | 0.006 | 0.020 | 0.7817 |
| | CDKN1A | Intercept | -0.53 | 0.46 | 0.2534 | -0.60 | 0.40 | 0.1374 |
| | | Age | -0.002 | 0.0001 | 0.0212 | 0.002 | 0.0009 | 0.0145 |
| Proliferation/antiproliferation | | Dose | -0.37 | 0.11 | 0.0017 | -0.10 | 0.00400 | 0.0040 |
| | SIRT3 | Intercept | -0.03 | 0.26 | 0.9180 | NS[a] | NS[a] | NS[a] |
| | | Age | -0.001 | 0.0006 | 0.0317 | NS[a] | NS[a] | NS[a] |
| | | Dose | 0.19 | 0.06 | 0.0028 | NS[a] | NS[a] | NS[a] |
| | PTGES | Intercept | -0.20 | 0.19 | 0.2971 | 1.19 | | <0.0001 |
| Paracrine signaling | | Age | -0.08 | 0.05 | 0.0059 | -0.002 | 0.0009 | <0.0001 |
| | | Dose | -0.001 | 0.0004 | 0.0774 | -0.10 | 0.04 | 0.0040 |

[a]NS  Non-significant. Assumptions violated for univariate model.

Units for age are days; units for dose are Gy. Parameters are for $\Delta\Delta C_t$
